# PDCG-Enhanced CNN for Pattern Recognition in Time Series Data

**DOI:** 10.3390/biomimetics10050263

**Published:** 2025-04-24

**Authors:** Feng Xie, Ming Xie, Cheng Wang, Dongwei Li, Xuan Zhang

**Affiliations:** 1 School of Information Science and Technology, Sanda University, Shanghai 201209, China; c.wang@sandau.edu.cn (C.W.);; 2 School of Mechanical and Aerospace Engineering, Nanyang Technological University, Singapore 639798, Singapore; mmxie@ntu.edu.sg

**Keywords:** similarity analysis, patterns identification, Fréchet distance, dynamic time warping, convolutional neural networks, pattern-driven case generators, time series

## Abstract

This study compares the effectiveness of three methods—Fréchet Distance, Dynamic Time Warping (DTW), and Convolutional Neural Networks (CNNs)—in detecting similarities and pattern recognition in time series. It proposes a Pattern-Driven Case Generator (PDCG) framework to automate the creation of labeled time series data for training CNN models, addressing the challenge of manual dataset curation. By injecting controlled noise and interpolating diverse shapes (e.g., W/M/nAn/vVv), a PDCG synthesizes realistic training data that enhances model robustness. Experimental results demonstrate that the CNN model, trained with 10,000 PDCG-generated cases, achieves 86–98% accuracy in pattern recognition, outperforming traditional methods (Fréchet and DTW) for complex, misaligned, and variable-length sequences. The PDCG-enhanced CNN’s scalability and adaptability improve with larger datasets, validating the PDCG’s efficacy in bridging simulation and real-world applications.

## 1. Introduction

Time series similarity and pattern analysis underpin critical real-world applications. For example, in healthcare, clinicians rely on ECG pattern recognition to diagnose arrhythmias, where distinct wave patterns correlate with specific cardiac conditions; financial institutions leverage time series pattern analysis to predict market trends and optimize inventory management based on historical sales data [1]. A similarity analysis of time series curves is also useful in indoor positioning systems. By treating WiFi Received Signal Strength Indicator (RSSI) values as a time series, certain locations may exhibit unique patterns of signal strength variations over time. Recognizing these distinctive patterns can help determine the location of a device within a building [2]. Time series trajectory or movement behaviors are optimized for the elderly in nursing homes, providing support for safety protection and emergency response, enabling the timely detection of abnormal behaviors and the implementation of appropriate measures.

The Fréchet Distance algorithm quantifies curve similarity by evaluating pointwise topological relationships, enabling precise comparisons [3]. This metric has been successfully deployed in applications such as driver–car trajectory matching and handwritten text classification [4,5]. Meanwhile, Dynamic Time Warping (DTW) serves as another established method for time series curve similarity analysis. Traditional approaches of DTW are interpretable but struggle with complex patterns, while deep learning models require prohibitively large, labeled datasets—a process that remains manual, time-consuming, and prone to inconsistencies [6,7].

Time series pattern recognition is crucial across domains, yet existing methods face significant limitations. While existing methods (DTW, Fréchet Distance) work for small datasets, deep learning alternatives require impractically large, labeled time series datasets—a well-documented challenge in recent years. Traditional similarity measures rely on manual feature extraction and domain expertise, unlike deep learning approaches (e.g., CNNs) that learn hierarchical representations automatically. However, current CNN applications lack labeled datasets for efficient training data generation. The current solutions face the issues of handling complex temporal patterns, minimizing labeling bottlenecks, and ensuring model interpretability.

Biological systems exhibit remarkable capabilities in processing temporal patterns. These natural phenomena are governed by evolutionary optimization, enabling organisms to adapt to dynamic environments through hierarchical feature extraction and noise tolerance—principles that inspire computational models for time series analysis. Morphological changes in natural systems highlight the role of generative variability in enhancing robustness [8,9]. Inspired by biological systems’ ability to generate diversity through mimicking natural noise tolerance and hierarchical feature extraction, observed in neural systems, our study proposes a Pattern-Driven Case Generator (PDCG) framework that mimics diversity generation through controlled noise injection and shape interpolation. A PDCG emulates biological systems’ robustness through controlled noise injection. Stochastic perturbations (e.g., Gaussian jitter in amplitude/timing) simulate environmental variability, akin to synaptic noise in neural systems that enhances fault tolerance during information processing.

This study explored similarity analysis with a specific focus on identifying patterns in time series data. The methodology leveraged Fréchet Distance, DTW, and CNNs, with CNNs being pivotal for handling intricate pattern recognition in sizable datasets. The core objective was to compare the efficacy and precision of these techniques in categorizing prototypical time series patterns. This study proposes a novel approach, a Pattern-Driven Case Generator (PDCG), to efficiently generate simulation data for training the CNN model. The PDCG framework is designed to emulate generative variability, where controlled noise injection mimics environmental perturbations and shape interpolation reflects adaptive morphological changes. This study aims to provide alternative perspectives and practical tools for data scientists concerning pattern detection, data analysis, and associated domains.

## 2. Background

The Fréchet Distance algorithm, as surveyed by Maheshwari and Yi (2018) [10], measures curve similarity by treating input curves as polylines and finding the path with minimized maximum pointwise distance. While it captures topological relationships between curve points for accurate similarity measurement, its high computational complexity and sensitivity to noise/time shifts limit its practical applications.

For time series alignment, Dynamic Time Warping (DTW) offers an alternative approach by optimizing point matching through time-axis warping under boundary, continuity, and monotonicity constraints. Though it shares the same O(nm) complexity as Fréchet Distance, DTW focuses solely on value similarity while ignoring point ordering. Despite its interpretability and temporal distortion handling, DTW struggles with high-dimensional data and shows no universal superiority across diverse datasets. Its computational cost also becomes prohibitive for long sequences.

Wang et al. (2013) [11] provided an empirical comparison of many time series representation methods and distance measures (e.g., Euclidean and DTW). Recent advances have expanded the methodological landscape. Wang et al. (2018) [12] introduced a framework integrating representation learning and kernel techniques; Franses and Wiemann (2020) [13] demonstrated economic time series applications; Abdelmadjid and Bachir (2021) [14] developed a long-series classification method combining local extrema with DTW; Agnieszka et al. (2022) [15] evaluated time series similarity using concept-based models; and Zhang et al. (2023) [16] proposed a time series similarity measurement method combining series decomposition with DTW.

Meanwhile, deep learning approaches (reviewed by Qahtan et al., 2020 [17]) automatically learn hierarchical features and complex relationships without manual feature engineering. While overcoming traditional methods’ limitations in handling high-dimensional noisy data, these neural network-based solutions require substantial training data and computational resources while suffering from interpretability challenges.

While deep learning models excel at automatically learning complex patterns without extensive parameter tuning, their deployment faces three key challenges: (1) data-intensive requirements—they typically need thousands to millions of labeled examples for robust training—(2) high computational costs due to resource-demanding architectures, and (3) limited interpretability arising from their “black-box” nature, which complicates troubleshooting ambiguous predictions.

The manual compilation of such voluminous labeled data can be enormously time-consuming and labor-intensive. To address this, this study proposes a simulation-based approach for efficiently generating labeled time series data for pattern recognition. Generating a spectrum of simulated training exemplars encoding complex morphologies enables more robust CNN model learning, as demonstrated by Shorten et al. in 2019 [18]. The ability to train deep networks bounteously was predicted using the proposed simulation methodology.

Our PDCG-enhanced CNN specifically leverages these bio-inspired principles through multiscale analysis inspired by Agnieszka et al. [15], combining synthetic data generation with deep learning’s pattern recognition strengths. Additionally, recent advances in hybrid deep learning models demonstrate superior performance for time series classification tasks. Batool et al. (2024) [19] developed a CNN-LSTM ensemble that achieved 3–5% higher accuracy than standalone models in wearable sensor applications, leveraging tailored data augmentation techniques. Similarly, Khan et al. (2025) [20] integrated CNNs with transformers for multimodal time series data (IMU, ECG, PPG), using attention mechanisms to dynamically weight sensor modalities and improve robustness to noise and missing data. These approaches exemplify the broader trend favoring hybrid architectures (e.g., CNN-LSTM, DTW-ANN) over single-model methods, as they capture both spatial and temporal dependencies more effectively. Both studies highlighted limitations in terms of computational overhead for the ensemble methods, and multimodal fusion restricts deployment on resource-constrained edge devices, while dependence on extensive labeled data remains a persistent challenge.

## 3. Materials and Methods

### 3.1. Typical Patterns

**Figure 1:**
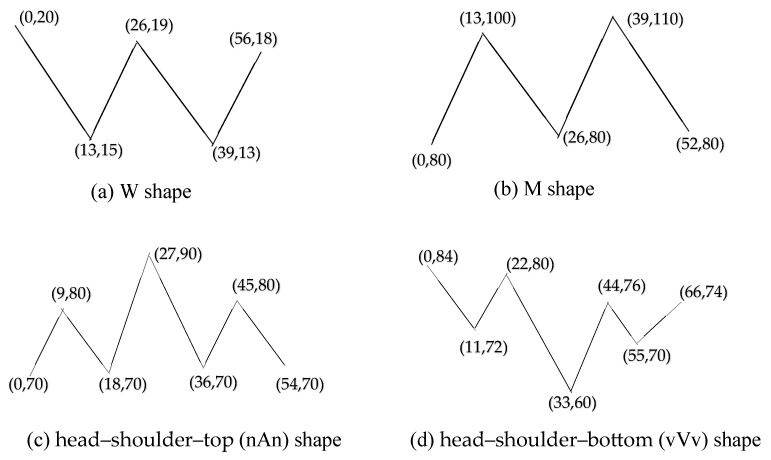
Definitions of four typical patterns (**a**–**d**).

Time series data exhibit distinct patterns depending on their source and application. For simplicity, four typical patterns were discussed in this study, denoted as W, M, nAn, and vVv shapes, which were defined in the coordinate system with their nodes specified by (x,y) values, as shown in Figure 1. The selection of W, M, nAn, and vVv as core patterns was driven by their morphological distinctness and real-world applicability. These shapes were designed to represent fundamental time series behaviors that are frequently observed in domains like healthcare and finance (e.g., W/M, oscillatory patterns, also common in sensor data; nAn/vVv, symmetric peaks/valleys in financial market data). The “Other” group ensured generalizability to unpredictable scenarios. This balanced approach—combining well-defined templates with a flexible residual category—ensured robustness in pattern recognition across diverse applications.

In Figure 1a, for example, a W shape is illustrated with the coordinates (0, 20), (13, 15), (26, 19), (39, 13), (56, 18); the other shapes are also indicated. W, M, and different types of shapes have differences in local peak and valley structure and time series dynamics.

Data normalization is essential for Fréchet and DTW algorithms. This study employed min–max normalization, which ensured that all features had the same scale.

### 3.2. Data Collection

**Figure 2:**
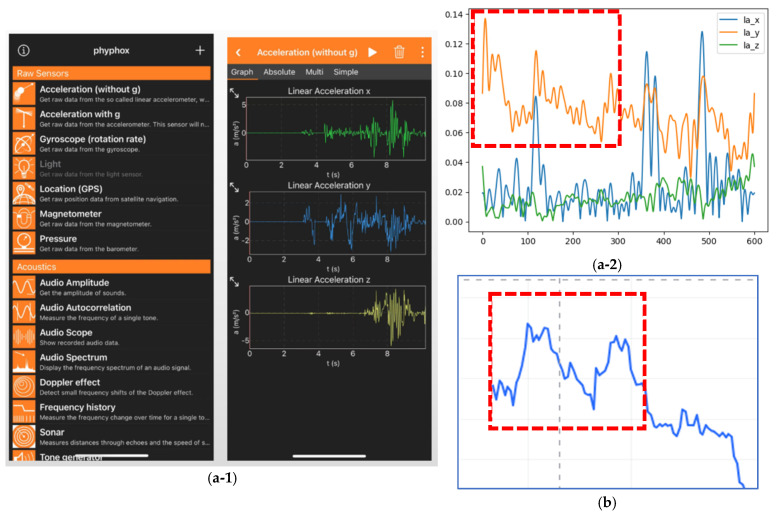
(**a-1**) Illustration of real cases. (**a-2**) W shape in real case (SensorData); (**b**) M shape in real case (FinData).

A total of 500 real test cases were collected from built-in smartphone sensors (linear accelerometers), as shown in Figure 2(a-1,a-2). Twenty students walked while holding their phones, performing gestures like the “shake” function or wobbling actions. Phyphox software (version 1.1.16) was used to record and export linear accelerometer data files. The data were sampled at a frequency of 50 Hz and the sampling time was set to ensure that each data collection session lasted for at least 30 s. This was in order to ensure a sufficient amount of data for analysis and to capture various movement patterns.

Another 500 real test cases were randomly selected from the domestic financial market, comprising 69 different commodities’ prices over the time range from 12 March 2015 to 15 January 2025, with Figure 2b showing an example.

### 3.3. Labeling Process

Each case was manually labeled with the shape categories W, M, nAn, vVv, and Other. For a time series to be labeled as a specific shape (W, M, nAn, or vVv), it had to closely match the predefined geometric characteristics of that shape. For example, a W-shaped pattern should have two local minima and one local maximum in a specific relative position and with certain slope characteristics between the points. If a time series did not clearly match any of these four typical shape categories, it was labeled as Other type.

After initial labeling by one student, a second student independently reviewed 20% of the labeled cases. Any discrepancies in labeling were discussed and resolved through a consensus-building process involving both students. If the disagreement persisted, a third student or lecturer in the field was consulted to make the final decision. This process ensured the accuracy and consistency of the labeling.

### 3.4. Pattern-Driven Case Generator (PDCG)

A method for a Pattern-Driven Case Generator (PDCG) was introduced to automatically generate simulation data for training the CNN model. The noise injection in the PDCG was inspired by biological systems’ inherent noise tolerance. The PDCG randomly generated simulated time series curves based on the specified pattern templates. It was designed using a set of linear formulas, automatically producing simulated time series curves with noise injections based on defining canonical shape templates, mathematical interpolation between points, and normalization.

**Figure 3:**
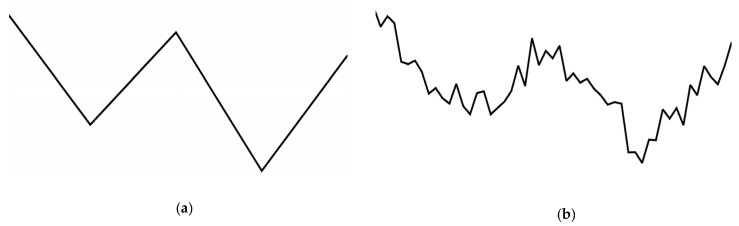
Example of Pattern-Driven Case Generator output for W-shaped pattern (**a**,**b**).

For example, given the typical W-shaped pattern defined in Figure 3a, its key features include five data points, with *X* (time) values of [0, 13, 26, 39, 56] and *Y* values of [20, 15, 19, 13, 18]. A point-slope equation was used to obtain the front and back coordinates of each segment. These line segments were then linked individually to construct the complete W-shaped formula, as shown in Equation (1), where *i* = 0, 1, 2, 3, …, *N*. Here, *N* is the total number of nodes in the time series curve.

(1) $$y=y_{i+1}+\frac{y_{i+1}-y_{i}}{x_{i+1}-x_{i}}*\left(x-x_{i}\right)$$

Subsequently, based on the horizontal *X* coordinates of each point, intervals were calculated between the subsequent points’ *X* coordinates. This allowed the determination of the corresponding *Y*-axis values at evenly spaced *X* coordinates between the defined points. Connecting these interpolated *Y* values resulted in a complete data sequence, forming a typical pattern. Finally, random noise was added to the generated data sequence to produce the simulated pattern-driven curves.

Figure 3 illustrates how the Pattern-Driven Case Generator worked to produce simulated time series curves with added noise based on specified canonical shape templates like a W-shaped pattern. In Figure 3a, a W shape was generated based on Equation (1). In Figure 3b, we show the simulated W-shaped curve generated from the pattern in (a) after the normalization of y values and the addition of random noise.

Random noise was added to simulate the random fluctuations in real data, enhance the diversity of the simulated data, and improve the model’s robustness to noise. The technical principle used was the superimposition of Gaussian noise on the time series generated by interpolation, and the noise intensity was controlled by the standard deviation σ. The mathematical model was as follows:

For each point *Y*, independent Gaussian noise is added:

(2) $$Y_{noisy}=Y+\varepsilon ,\varepsilon \sim N(0,\sigma^{2})$$

where *ε* follows a normal distribution with a mean of 0 and a variance of *σ*^2^.

In the above example, the simulated *y* values are [1.09, 0.9, 0.95, 0.88, 0.82, 0.79, 0.65, 0.68, 0.57, 0.53, …, 0.39, 0.48, 0.36, 0.39, 0.5, 0.6, 0.64, 0.64], with a total of 41 data points.

**Figure 4:**
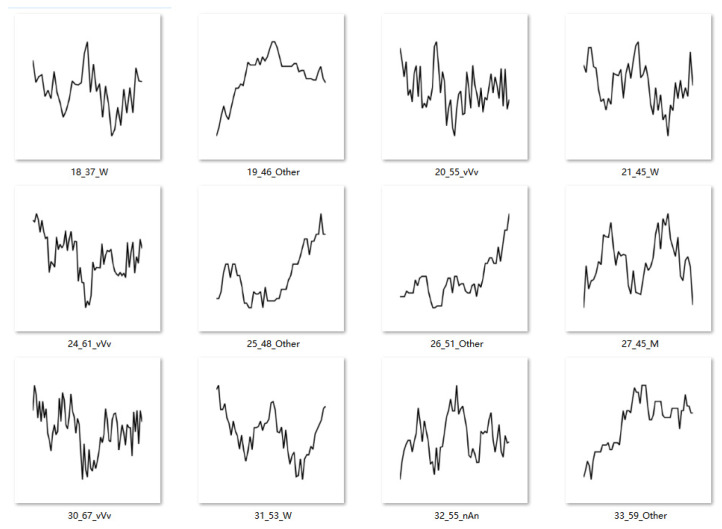
Examples of pattern-driven simulation test cases.

In the experiment, 10,000 simulated cases were generated with labeled correct typical patterns, as shown in Figure 4. Among the 10,000 cases, apart from the four typical patterns mentioned in Figure 1, another type of curve was also randomly generated labeled as the “Other type” for future classification, based on the following algorithm:

Step 1: Initialization:

Set *N* = the total number of nodes, random integer between 20 to 130;

Set *X* = [0, 1, 2, 3, …, *N*];

Set *Y* = [*Y*_0_], *Y*_0_ was a random value between −100 and 100.

Step 2: For *i* from 1 to *N*:

Let random_walk = random number between −1 and 1;

Let scale = random number between 1 and 10;

Let step = random_number × scale;

*Y_i_* = *Y_i_*_−1_ + step, append (*Y_i_*) to *Y.*

Step 3: Normalize *Y_i_* to [0, 1].

Step 4: Repeat and iterate over all *Y_i_*.

### 3.5. Framework

**Figure 5:**
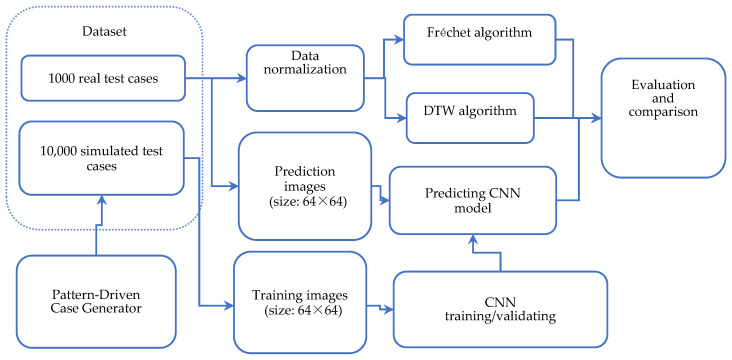
Overall study framework and methods.

For the CNN identification of all the simulation curves, all the simulation cases were transformed into 64 × 64 pixel grayscale JPEG images. Google TensorFlow was used to train and validate the CNN model. TensorFlow Keras, a high-level API on the TensorFlow platform, provided an intuitive, highly productive interface for solving machine learning (ML) problems with a focus on modern deep learning. Figure 5 illustrates the overall study framework.

## 4. Experiments

### 4.1. Dataset

As listed in Table 1, a dataset was prepared for the experiment. The training dataset for the CNN model was derived from the simulated cases. Datasets containing 200, 400, …, 8000, and 10,000 simulation cases were generated repeatedly for each training. The aim was to investigate the impact of the dataset size on the model’s performance. For each dataset size, the data were split, with 60% used for training the CNN and 40% reserved for validation.

In Table 1, the first column (“Simulation Cases”) represents the different dataset sizes used for training the CNN model, ranging from 200 to 10,000 simulated cases. These sizes were chosen in order to analyze how increasing training data volume impacted model accuracy, as seen in the experiment design. The columns under “Patterns (Four Typical Types and “Other” Type)” indicate the number of cases per pattern type in each dataset. The decimal values (e.g., 47.47 (5.2)) represent the mean length (N) of the time series data points (e.g., 47.47) and standard deviation (e.g., 5.2), showing the variability in length.

A total of 500 real test cases were collected and selected from built-in mobile smartphone sensors (liner accelerometers), denoted as SensorData. Another 500 real test cases were randomly selected from the domestic financial market, denoted as FinData. A description of the 1000 real cases is given in Table 2.

This study employed data normalization to ensure that all features had the same scale. Min–max normalization (0–1 range) was applied to account for scaling differences.

### 4.2. Test Scenarios

Various test scenarios were created to test and compare the effectiveness of the methods. These scenarios included different combinations of W, M, nAn, vVv, and Other shape patterns, as outlined in Table 3. The main objective was to assess each method’s ability to accurately differentiate between different patterns by combining the first four shape categories across multiple test cases.

## 5. Results

The time series datasets from the simulation cases were transformed into grayscale JPEG images with dimensions of 64 × 64 pixels. These images were then read using the OpenCV Python (Version 3.11) library and converted into NumPy arrays. To ensure consistency, the matrix input data X were normalized by dividing each element by 255. TensorFlow Keras was used to train the CNN model. The structure of the model is shown in Table 4.

The CNN model was compiled using the Adam optimizer and SparseCategoricalCrossentropy loss function. SparseCategoricalCrossentropy is a loss function used for multiclass classification problems in deep learning models such as neural networks. The convolutional layers utilized 64 filters with kernels, followed by max pooling, and the model architecture included three convolutional blocks before flattening and two dense layers (see Table 4). For model training, structured hyperparameter searches like grid search for critical parameters (e.g., learning rate) and random search for architectural choices were implemented. Finally, the model adopted the Adam optimizer with a learning rate of 0.001 and the batch size was set to 32. The model was trained for 100 epochs with early stopping (patience = 30) to halt training if validation performance plateaued, ensuring computational efficiency while maintaining model accuracy.

These experiments were carried out to verify and compare the Fréchet, DTW, and CNN methods for time series curve similarity. Through experimentation, it was found that all three methods did not require the time series curves to be aligned on the time axis and could handle time series curves of different lengths. The CNN learning model could tolerate anomalies and distortions in all types of time series curves and obtained more accurate results than the Fréchet and DTW algorithms.

The results presented in Table 5 demonstrate the validity of both the Fréchet and DTW algorithms in accurately categorizing the test cases from Scenario 1 and 5. Scenario 1 consisted of 200 real cases split between two shape categories: W and M shapes. The DTW algorithm correctly distinguished between these two categories with accuracies of 97% and 98% (values adjacent to the (|) are based on real cases in SensorData and FinData, respectively). Comparatively, the Fréchet algorithm achieved 86% and 79% accuracy in categorizing the test cases of these two shape types. This evidence supports the superior validity of the DTW algorithm over the Fréchet algorithm for this classification task.

**Figure 6:**
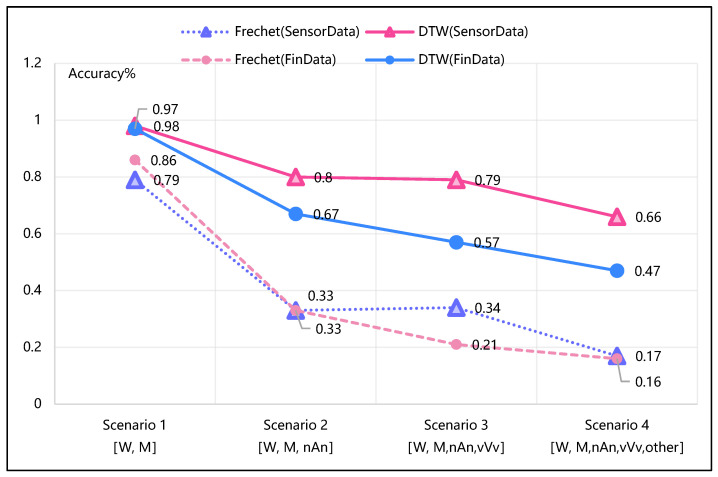
Accuracy comparison of Fréchet and DTW algorithms with increasing pattern complexities (2 to 5 shapes).

Figure 6 shows the accuracy achieved by the Fréchet and DTW algorithms in identifying different combinations of time series pattern shapes across test Scenarios 1 through 4. The *x*-axis lists the test scenario IDs, each representing a different set of pattern shapes to be distinguished. The *y*-axis shows the accuracy values. This highlights how the accuracy of both algorithms degraded when more pattern categories, especially the random “Other” shape, were introduced.

Referring to Figure 6, in test Scenario 1 (200 real cases, [W, M]), only two shapes are observed (W and M). Fréchet accuracy declines sharply (from 79–88% to 16–17%) with the inclusion of the “Other” category (Scenario 4), while DTW shows higher robustness but still experiences a significant drop (from 97–98% to 47–66%). As the number of shape categories increases, particularly with the addition of a random/arbitrary shape, the accuracy of both algorithms declines.

**Figure 7:**
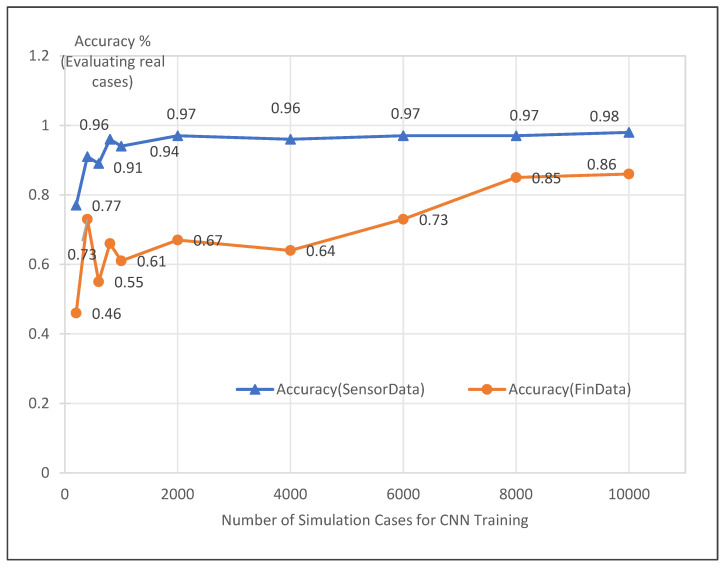
Impact of simulated training dataset size on CNN accuracy for 5-shape classification.

In Figure 7, we show how the accuracy of the CNN model for the 500 real test cases (test Scenario 5 with all 5 pattern shapes [W, M, nAn, vVv, Other]) changed as the size of the simulated training dataset increased. The *x*-axis represents the different sizes of the simulated training datasets used, ranging from 200 to 10,000 cases. The *y*-axis shows the accuracy values. The plot demonstrates that as more simulated training data was provided to the CNN, allowing it to learn from a larger variety of examples, its accuracy on the real test cases improved substantially. The accuracy increased to 86–98% as the training set size reached 10,000 simulation cases.

As the size of the simulated training dataset increases, the CNN model has more diverse examples to learn from. This allows the model to better capture the features and patterns in time series data, leading to improved accuracy. This increase in accuracy can be attributed to the model’s ability to generalize better with more training data.

## 6. Conclusions

This study conducted an analysis of time series similarity, focusing on identifying typical patterns in time series data using Fréchet Distance, DTW, and a CNN. A novel approach, a Pattern-Driven Case Generator (PDCG), was introduced to generate simulation data to efficiently train the CNN model. By integrating bio-inspired diversity generation (via the PDCG) and hierarchical learning (via a CNN), this study bridges computational models with neural network principles, offering a novel paradigm for time series analysis. The study meticulously compared the performance of the Fréchet Distance, DTW, and CNN models in categorizing complex time series patterns such as W-, M-, nAn-, vVv-shaped, and others.

Through experimentation, it was found that all three methods do not require the time series curves to be aligned on the time axis and can handle time series curves of different lengths. The study revealed nuanced performances across the Fréchet Distance, DTW, and CNN models. CNN models demonstrate superior adaptability and accuracy, particularly with extensive training data, reaching 86–98% accuracy with 10,000 simulation cases for training. The Fréchet model achieved 79–88% accuracy, while DTW achieved 97–98% accuracy in handling time series curves with W- and M-shaped curves. However, expanding to five shapes sharply reduced the Fréchet model’s accuracy to 17%, while the DTW accuracy dropped significantly to 47–66%. As the number of shape categories increased, particularly with the addition of a random/arbitrary shape, the accuracy of both algorithms declined.

This study highlighted the novel use of a PDCG for simulation data generation, which is pivotal for CNN training. It could distinguish well between all the different types of patterns using simulation data produced by the PDCG for different real-world scenarios. With 10,000 training simulation cases, the PDCG-enhanced CNN achieved 86–98% accuracy, significantly outperforming Fréchet and DTW, validated across synthetic and real datasets. This innovative approach enhances the training process, allowing the CNN to better learn and identify various time series patterns. This reveals that the CNN’s adaptability and accuracy are partly due to the diverse and large dataset provided by the PDCG, which is crucial for deep learning models. The relationship between dataset quality and model performance is a critical finding of this study.

While the study demonstrates the CNN’s superior accuracy when trained with PDCG-generated data, there are several limitations, highlighted as follows. First, computational costs remain a challenge, as CNNs demand significant resources, particularly with large-scale simulated datasets. Second, risks of overfitting persist if the training data lack diversity in noise or edge-case patterns, necessitating techniques like dropout or adversarial validation. The inherent “black-box” nature of CNNs limits their interpretability, particularly for ambiguous patterns such as those categorized under “Other”. To address this critical challenge for real-world adoption, enhancing model transparency through techniques like feature visualization, LIME, or attention mechanisms will be prioritized in future work.

While the current study employed a 60/40 train–validation split to evaluate model performance across varying dataset sizes, we acknowledge the need for more rigorous validation techniques. We need to implement cross-validation and ablation study in future iterations to ensure robust performance estimation and mitigate overfitting risks.

Additionally, while the study addresses the issue of noise in the simulation data through Gaussian noise injection, it does not delve deeply into real-world challenges like missing data, outliers, or irregular sampling rates, which are common in time series data. While the PDCG enhances data diversity, real-world complexities (e.g., non-Gaussian noise, missing values) may not be fully captured, requiring validation on broader datasets. Future work should prioritize efficiency, interpretability, and robustness testing under adversarial conditions to ensure real-world applicability, and address more real-world challenges (missing data, irregular sampling) via synthetic data augmentation as well. Beyond immediate performance gains, the PDCG framework needs to further enable the rapid generation of domain-specific training data—particularly valuable for fields like medicine where labeled data are scarce. Future work should explore transfer learning to adapt our pretrained models to niche applications while maintaining computational efficiency.

## Figures and Tables

**Table 1:** Information of simulation cases.

Simulation Cases	Patterns (Four Typical Types and “Other” Type)				
	W	M	nAn	vVv	Other
200 ^1^	38 ^2^	37	36	39	50
	47.47 (5.2) ^3^	48.11 (4.96)	70.5 (8.17)	72.31 (8.59)	50.72 (5.96)
400	95	83	81	79	62
	48.67 (5.01)	48.58 (5.8)	71.11 (8.23)	71.24 (8.23)	50.26 (6.01)
600	130	118	127	117	108
	48.74 (5.72)	47.49 (5.86)	71.39 (8.17)	71.28 (8.56)	49.5 (6.29)
800	156	162	162	154	166
	47.77 (5.82)	47.88 (5.99)	72.56 (8.45)	71.57 (7.86)	50.19 (5.56)
1000	203	188	180	211	218
	48.24 (5.64)	48.04 (5.69)	72.27 (8.14)	71.09 (8.64)	48.79 (5.53)
2000	375	417	376	403	429
	48.28 (5.74)	47.7 (5.6)	72.51 (8.57)	72.04 (8.7)	49.56 (5.8)
4000	802	777	787	843	791
	48.1 (5.77)	48.08 (5.76)	71.97 (8.55)	72.46 (8.6)	49.64 (5.77)
6000	1174	1224	1121	1250	1231
	48.09 (5.61)	48.1 (5.58)	71.89 (8.48)	71.6 (8.39)	49.49 (5.88)
8000	1651	1582	1595	1621	1551
	48.09 (5.66)	47.98 (5.61)	72.12 (8.45)	71.81 (8.51)	49.37 (5.89)
10,000	1992	1975	1960	2052	2021
	48.08 (5.61)	48.31 (5.71)	72.02 (8.43)	71.76 (8.32)	49.63 (5.68)

^1^ The number of simulation cases used for different trainings of the CNN model. ^2^ The mean sizes of the simulation cases with the specified patterns. ^3^ The mean (standard deviation) values for the lengths (*N*, as described in Section 3) of the time series.

**Table 2:** Description of real cases.

Real Cases with Diff. Patterns	W	M	nAn	vVv	Other
**Data source: Phone sensors (SensorData)**					
Size of real cases	100	100	100	100	100
Types of data involved	Linear acceleration data (*x*, *y*, *z* axes) from smartphone sensors				
Size of time series (noted as *N* ^1^)	Minimum: 35; maximum: 65				
Mean of N	51.45	55.90	57.20	50.50	45.45
Std. of N	16.3	15.1	16.1	12.2	19.4
**Data source: Financial market (FinData)**					
Size of real cases	100	100	100	100	100
Number of commodities with prices	44	40	39	37	35
Size of time series (noted as *N* ^1^)	Minimum: 20; maximum: 130				
Mean of N	71.4	70.8	80.6	81.2	75.1
Std. of N	28.7	26.8	30.3	29.6	27.5

^1^ *N* is the length of the time series, as described in Section 3.

**Table 3:** Description of test scenarios for evaluation.

Test Scenario ID	Method	Patterns to Be Distinguished	Number of Simulation Cases for Training CNN	Number of Real Cases for Overall Evaluating
1	Fréchet, DTW	[W, M]	NA	200
2	Fréchet, DTW	[W, M, nAn]	NA	300
3	Fréchet, DTW	[W, M, nAn, vVv]	NA	400
4	Fréchet, DTW	[W, M, nAn, vVv, Other]	NA	500
5	CNN	[W, M, nAn, vVv, Other]	200, 400, …, 8000, 10,000, respectively	500

**Table 4:** CNN model structure.

Layer (Type)	Output Shape	Num. of Parameters
conv2d (Conv2D)	(None, 62, 62, 64)	640
max_pooling2d (MaxPooling2D)	(None, 31, 31, 64)	0
conv2d_1 (Conv2D)	(None, 29, 29, 64)	36,928
max_pooling2d_1 (MaxPooling2D)	(None, 14, 14, 64)	0
conv2d_2 (Conv2D)	(None, 12, 12, 64)	36,928
max_pooling2d_2 (MaxPooling2D)	(None, 6, 6, 64)	0
conv2d_3 (Conv2D)	(None, 4, 4, 64)	36,928
flatten (Flatten)	(None, 1024)	0
dense (Dense)	(None, 64)	65,600
dense_1 (Dense)	(None, 5)	325

**Table 5:** Accuracy results for Fréchet, DTW, and CNN.

	Precision ^1^	Recall ^2^	f1-Score ^3^	Support ^4^
**Test Scenario 1: 200 real cases for evaluating, [W, M]**				
**Fréchet**				
W	0.8 | 0.75 ^5^	0.96 | 0.88	0.87 | 0.81	100
M	0.95 | 0.85	0.76 | 0.70	0.84 | 0.77	100
**accuracy**			0.86 | 0.79	200
**macro avg**	0.88 | 0.79	0.88 | 0.79	0.88 | 0.79	200
**weighted avg**	0.88 | 0.79	0.88 | 0.79	0.88 | 0.79	200
**DTW**				
W	1.00 | 0.98	0.94 | 0.98	0.97 | 0.98	100
M	0.94 | 0.98	1.00 | 0.98	0.97 | 0.98	100
**accuracy**			0.97 | 0.98	200
**macro avg**	0.97 | 0.98	0.97 | 0.98	0.97 | 0.98	200
**weighted avg**	0.97 | 0.98	0.97 | 0.98	0.97 | 0.98	200
**macro avg**	0.97 | 0.98	0.97 | 0.98	0.97 | 0.98	200
**Test Scenario 5:** **10,000 simulation cases for CNN training, 500 real cases for evaluating, [W, M, nAn, vVv, Other]**				
**CNN**				
W	0.98 | 0.79	0.99 | 0.93	0.99 | 0.86	100
M	0.99 | 0.96	0.99 | 0.95	0.99 | 0.95	100
nAn	0.99 | 0.96	1.00 | 0.71	1.00 | 0.82	100
vVv	0.96 | 1.00	1.00 | 0.74	0.98 | 0.85	100
Other	1.00 | 0.70	0.94 | 0.95	0.97 | 0.81	100
**accuracy**	0.98 | 0.86	0.98 | 0.86	0.98 | 0.86	500
**macro avg**	0.98 | 0.88	0.98 | 0.86	0.98 | 0.86	500
**weighted avg**	0.98 | 0.88	0.98 | 0.86	0.98 | 0.86	500

^1^ Precision: The ratio of true positives to the sum of true and false positives. ^2^ Recall: The ratio of true positives to the sum of true positives and false negatives. ^3^ f1-Score: A weighted harmonic mean of precision and recall such that the best score is 1.0 and the worst is 0.0. ^4^ Support: The number of actual occurrences of the class in the specified dataset. ^5^ Values adjacent to the | are based on real cases in SensorData and FinData, respectively.

## Data Availability

Data are contained within the article.
